# Use of biomass fuels predicts indoor particulate matter and carbon monoxide concentrations; evidence from an informal urban settlement in Fort Portal city, Uganda

**DOI:** 10.1186/s12889-022-14015-w

**Published:** 2022-09-12

**Authors:** Winnifred K. Kansiime, Richard K. Mugambe, Edwinah Atusingwize, Solomon T. Wafula, Vincent Nsereko, Tonny Ssekamatte, Aisha Nalugya, Eric Stephen Coker, John C. Ssempebwa, John Bosco Isunju

**Affiliations:** 1 Department of Disease Control and Environmental Health, School of Public Health, College of Health Sciences, Makerere University, P. O. Box 7072, Kampala, Uganda; 2 Department of Environmental and Global Health, College of Public Health and Health Professions, University of Florida, 1225 Center Drive, Room 4160, P. O. Box 100188, Gainesville, FL 32610 USA

**Keywords:** Charcoal, Outdoor cooking, Indoor air quality, Pollution

## Abstract

**Background:**

Poor indoor air quality (IAQ) is a leading cause of respiratory and cardiopulmonary illnesses. Particulate matter (PM_2.5_) and carbon monoxide (CO) are critical indicators of IAQ, yet there is limited evidence of their concentrations in informal urban settlements in low-income countries.

**Objective:**

This study assessed household characteristics that predict the concentrations of PM_2.5_ and CO within households in an informal settlement in Fort Portal City, Uganda.

**Methodology:**

A cross-sectional study was conducted in 374 households. Concentrations of PM_2.5_ and CO were measured using a multi-purpose laser particle detector and a carbon monoxide IAQ meter, respectively. Data on household characteristics were collected using a structured questionnaire and an observational checklist. Data were analysed using STATA version 14.0. Linear regression was used to establish the relationship between PM_2.5,_ CO concentrations and household cooking characteristics.

**Results:**

The majority (89%, 332/374) of the households used charcoal for cooking. More than half (52%, 194/374) cooked outdoors. Cooking areas had significantly higher PM_2.5_ and CO concentrations (t = 18.14, *p* ≤ 0.05) and (t = 5.77 *p* ≤ 0.05), respectively. Cooking outdoors was associated with a 0.112 increase in the PM_2.5_ concentrations in the cooking area (0.112 [95% CI: -0.069, 1.614; *p* = 0.033]). Cooking with moderately polluting fuel was associated with a 0.718 increase in CO concentrations (0.718 [95% CI: 0.084, 1.352; *p* = 0.027]) in the living area.

**Conclusions:**

The cooking and the living areas had high concentrations of PM_2.5_ and CO during the cooking time. Cooking with charcoal resulted in higher CO in the living area. Furthermore, cooking outdoors did not have a protective effect against PM_2.5_, and ambient PM_2.5_ exceeded the WHO Air quality limits. Interventions to improve the indoor air quality in informal settlements should promote a switch to cleaner cooking energy and improvement in the ambient air quality.

**Supplementary Information:**

The online version contains supplementary material available at 10.1186/s12889-022-14015-w.

## Background

Globally, indoor air pollution (IAP) was responsible for 3.8 million death in 2018 [1], contributing to 8% of the global mortality [2] and 91.5 million disability-adjusted life years (DALYs) [3] in 2019. It represented the third leading risk factor (6% of global DALYs) among children under five years and the second leading risk factor in disease burden for women globally [4]. The public health threat of IAP is highest in low-and middle-income countries ((LMICs), where it contributes to approximately 10% of mortality [2], resulting in a 1000-fold difference from high-income countries [5]. In 2019, household air pollution (HAP) resulted in 697,000 deaths in Africa [6]. IAP is significantly associated with respiratory tract infections in Uganda, especially in children [7, 8].

Household air pollution is generated by various sources such as tobacco smoking, outdoor sources, and combustion of fuels, among others. Incomplete combustion of fuel leads to IAP with emission of air pollutants, including fine nitrogen dioxide (NO_2_), particulate matter (PM) and CO [9, 10]. The widespread use of solid fuel combustion for cooking and heating energy needs among an estimated 3 billion people in LMICs is partly responsible for household air pollution. Reliance on solid fuels for household energy in LMICs is mainly due to limited access (availability and affordability) to cleaner sources of energy such as electricity or liquefied petroleum gas (LPG) [11]. The number of people using solid fuels for household energy needs is expected to increase through 2030, and Sub-Saharan Africa is projected to have the highest increase in household solid fuel use for cooking [12, 13]. This may result in HAP due to the rapid urbanisation and unmitigated household use of solid fuels.

Available data indicate that 90% of households in Uganda use solid biomass fuel, which elevates the risk of household air pollution [14]. Inhalation of air pollutants such as PM leads to the development of cardiopulmonary illnesses, while very high CO exposure is associated with hypoxia that affects organs with increased oxygen consumption, including the developing fetus [15–17]. Furthermore, exposure to PM_2.5_ and CO is associated with acute lower respiratory infections (ALRI), which are estimated to cause 5,700 deaths in children in Uganda annually [8, 18]. The risk is exacerbated in crowded urban environments such as informal settlements since urban environments are responsible for producing 78% of carbon emissions [19]. In addition, informal settlements are characterised by crowded, dilapidated and unregulated housing structures [20]. These generally have poor ventilation, inadequate water, sanitation and hygiene access, limited services and infrastructure, and low government response to needs and services [21]. Furthermore, the settlements lack planned and allocated cooking areas and have other unmitigated sources of air pollution such as open burning of solid wastes [22] and poor ambient air quality [23]. These conditions lead to the “triple threat” of communicable, non-communicable diseases (NCDs) and injuries in these informal urban settlements. In addition, informal settlements in Uganda are at risk of increased rural–urban migration due to the anticipated urbanisation of the cities [24], resulting in population growth and an increase in demand and usage of cooking fuel energy.

To combat air pollution in Uganda, the Ministry of Energy and Mineral Development instituted a Value added tax (VAT) waiver [24] of 17% on LPG. It embarked on national grid expansion and reinforcement for electricity [25] to promote the usage of cleaner LPG and electricity for cooking. Additionally, the Ministry of Lands, Housing and Urban Development oversee the physical planning of areas in Uganda and regulate and approve building plans for each district to reduce informal settlements and unregulated structures. Despite these measures, IAP is still prevalent [26]. Although there is evidence of IAQ and associated health effects and environmental risks such as exposure levels in rural and urban formal settings [8, 27–29], a few studies have been conducted in informal urban settlements, where a vast majority of the urban population in low-income countries reside [30].

In addition, the established IAQ guidelines cater for a 24 h average [11] which includes both cooking and non-cooking time, from which IAQ has been found to differ significantly [31]. Furthermore, the predictors of PM_2.5_ and CO concentrations in the cooking and the living area of informal dwellings during the cooking time remain inadequately explored. Therefore, this study aimed to assess household characteristics that predict the concentrations of PM_2.5_ and CO within households in Kisenyi-Mugunu, an informal settlement in the newly created city of Fort Portal city, Uganda.

## Methods

### Study setting and population

**Fig. 1:**
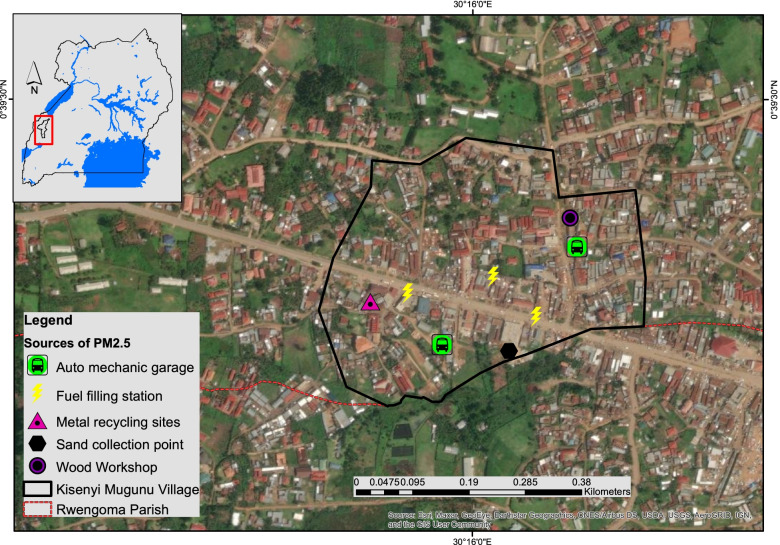
A map showing Kisenyi-Mugunu and sources of PM_2.5_

The study was conducted in Kisenyi-Mugunu, an informal settlement in the Western division of Fort Portal City (Fig. 1). Fort Portal is the city of Kabarole district, located in Western Uganda. Kabarole district has a total population of 469,236. Of this population, 17% are children aged 0–4 years. In this district, only 18% of the population has access to electricity, with 63% using kerosene lamps for lighting [32]. Most residents have low-socioeconomic status and rely primarily on charcoal and firewood for cooking fuel. The housing structures in this area are generally informal and unregulated, with poor ventilation. There is also poor storage of charcoal which may lead to wetting when it rains, thus deteriorating the quality and cooking efficiency leading to increased smoke production when burnt [33]. The study population included residents of Kisenyi-Mugunu, and the study units were households in Kisenyi-Mugunu, Fort portal.

### Study design, sample size and sampling

A cross-sectional study design was used. Data were collected from the 10^th^ to the 19^th^ of September 2020. The required sample size was calculated using the Kish Leslie formula for cross-sectional studies [34]. A *p*-value of 50% was used due to limited evidence on IAQ in households in informal settlements and a 95% level of confidence with a margin of error of 0.05. Substituting into the formula translated to a minimum sample of 385 households. However, 11 households were dropped due to missing air quality measurements leaving us with 374 participants.

With the help of the village chairperson, households in Kisenyi-Mugunu that had children under five years of age were identified, and a list of these residents was provided to researchers. Study households were then randomly selected using computer-generated random numbers. Since this research is part of a larger study investigating the health effects of IAP on child respiratory health, inclusion criteria included having a child under five years and the caretaker of the child consenting to participate in the study. Exclusion criteria included having a very sick child. Respondents included caretakers of under-fives.

### Data collection

After the consenting process, household interviews were conducted using pretested structured questionnaires. The structured interview collected information on socio-demographic characteristics, type of fuel used for cooking, location of cooking area, number of meals cooked per day, duration spent cooking per day, fuel storage area, and the fuel cost. Additionally, observation checklists were used to establish the conditions related to cooking inside and around the home and the cooking practices of the household. The data collection tools were developed and validated by air quality experts based at the Makerere University School of Public Health. The translated household data collection tool was validated through preliminary pilot testing and subsequent revisions. Real-time photometric measurement of PM_2.5_ and humidity was done using a multi-purpose laser particle detector LKC-1000S + (Temtop, USA). This detector is equipped with a laser particle sensor, and its operating environments include a temperature range: 0–50 °C; relative humidity range: 0–90%; atmospheric pressure: one atm; PM_2.5_ measurement range: 0–999 µg/m^3^ with a resolution of 0.1 µg/m^3^. The time resolution is one min. The laser sensor used in this detector (Temtop LKC-1000S +) was evaluated in the laboratory and the field with the Federal Equivalent Method (FEM)-Grimm as the standard [35, 36]. Three readings average within one minute was recorded per measurement. Low intra-model variability (~ 7%) has been reported for this detector in field tests [36]. The same sensor was used for the entire study, which minimised variability within different households [35, 36]. Neither FEM-GRIMM nor Tapered Element Oscillating MicroBalance (TEOM) were available, so no detector calibration was done against them. Therefore, the monitor was left to rest overnight for 8 h in a ventilated room. After this calibration, it was kept in the room airtight for 10 min before each field trip.

Real-time measurements of CO and temperature were done using EXTECH Carbon Monoxide Meter Model CO15. This detector has an electrochemical sensor, and its operating environments include a CO measurement range: 0–999 ppm with a resolution of 1 ppm. The accuracy of the detector is 0 ~ 100 ppm: ± 20%, for 100 ~ 500 ppm: ± 15%. The accuracy for ambient conditions was 20 °C ± 5 °C (68°F ± 9 °F) and 50% relative humidity ± 20% relative humidity. The instrument was calibrated prior to the training and field measurements, and the default calibration point of 0 ppm was used. A three readings average within one minute, was recorded per measurement. For this study, IAQ measurements for both the cooking area and the living space were conducted during the cooking hours between 8:00 AM and 6:00 PM.

All 374 households were sampled only once in the cooking area and living space. Measurement was done after the cooking fuel had been lit and active cooking was taking place. The study had one PM_2.5_ monitor and one CO monitor available. Therefore, three readings were taken for one household within one minute, and the average was taken as the household reading for PM_2.5,_ CO, temperature and humidity. On average, 37 households were measured per day. The air samples were taken one metre above the floor (the approximate breathing zone height of a child under five years) and one metre from the cooking area (the approximate distance of a child under five years away from the cooking area). The monitors were placed with the air receivers/inlets at least 1.5 m away from the windows and the doors because of reduced airflow near surfaces [37]. One research assistant took the air quality reading, and the data were later entered into Kobo collect form for the corresponding household. This study was performed in the wet season when ambient air pollution is expected to be low [23]. The limitation of this study included i)it had only one PM_2.5_ monitor and one CO monitor available, therefore three readings average within one minute, was recorded per measurement for each household during cooking time; ii) the state of the ventilation of the widows/doors being opened or closed when the AQ measurements were not recorded while the actual AQ measurements were being taken; iii) the local meteorological conditions experienced during the sampling campaign were not recorded.

### Study variables

The dependent variables were the concentrations of PM_2.5_ and CO. The independent variables included the main type of fuel used for cooking, nature of kitchen ventilation, location of the cooking area, location of the fuel storage area, duration of cooking, usage of damp/wet fuel, and type of cookstove, and its state of repair. The main type of cooking fuels included 1) straw/shrubs/grass, 2) wood, 3) charcoal, 4) kerosene, 5) electricity and 6) LPG/cylinder gas was used. For inferential statistics, the main type of cooking fuel was re-categorised into three classes; 1) less polluting fuels (electricity, LPG and kerosene), 2) moderately polluting fuels (charcoal), and 3) highly polluting fuels (wood, straw, shrubs and grass). Adequate ventilation was defined as having two or more ventilation openings so placed as to ensure parallel or cross-ventilation [38]. Cooking outdoors was also considered adequate ventilation. The location of the cooking area included 1) inside the house, 2) outdoor, and 3) separate building outside the house. Outdoor cooking included cooking outside in the open yard with no over-structure or surrounding structure and outside on the veranda with either a roof above or none. The location of fuel storage area included 1) inside the house, 2) outdoor, and 3) separate building outside the house. The type of cookstove included the traditional cookstove and the modern cookstove, while the state of repair was defined as good- no visible or a few cracks/defects or poor—many cracks/defects that may/do influence cooking [39]. Therefore, cooking and living areas with two or more openings were considered to have adequate ventilation.

### Quality control, data management and statistical analysis

The study investigators conducted a two-day training of research assistants to enhance data quality. The pretest fieldwork was conducted in Bwaise II village, Kawempe division Kampala. This was purposively selected for the pretest because it had similar characteristics (informal and densely populated) to the study area. Research assistants were asked for feedback about the questions’ clarity and instructions’ effectiveness, and necessary revisions were made. Data were collected using the KoboCollect mobile application preloaded on mobile smartphones and tablets. Participant responses were entered in an offline Kobo collect form for each household. Data were submitted to a secure online server (www.kobo.humanitarianresponse.info) daily. The investigators conducted daily data quality checks. Only the study investigators had the security key to ensure data security. Data were downloaded into Microsoft Excel 2010 and exported to Stata 14.0 (StataCorp Texas, USA) for statistical analysis. Some participants’ responses were dropped due to missing air quality measurements leaving 374 participants. Data were analysed using both descriptive and inferential statistics. For the descriptive statistics, frequencies and cross-tabulations were generated (where appropriate). For the inferential statistics, linear regression was used to derive associations (β-beta coefficients) between PM_2.5_ and CO concentrations and household characteristics. The unpaired t-test was used to estimate the statistical significance of differences between PM_2.5_ and CO in the cooking and living area. The concentration of the PM_2.5_ and CO were log-transformed before running regressions for a more near symmetrical distribution. A variable with a *p*-value less than 0.05 was considered significant.

### Ethical considerations

Ethical approval for the study was obtained from Makerere University School of Public Health Higher Degrees Research and Ethics Committee (Reg No. 783). The study was also registered with Uganda National Council for Science and Technology (UNCST) (Registration number HS695ES). Administrative clearance was sought from the Kabarole district Local government, which presides over the study area. Information sheets and consent forms were available in the local language (Rutooro) or English with details on the purpose of the project, procedures to be followed and the risks and benefits of participation. Informed written consent to participate in the study was sought from all study participants and from their legal guardian(s) where appropriate. For illiterate participants, consenting was conducted in the local language (Rutooro) in the presence of a witness and confirmed by the participant’s thumbprint on the written consent form. The study was carried out in accordance with relevant guidelines and regulations under strict COVID-19 guidelines as provided by the government of Uganda and UNCST.

## Results

### Socio-demographic characteristics

A total of 374 respondents were interviewed, representing a response rate of 97%. The mean age of the respondents was 30.22 (SD ± 0.51), 95% CI [29.21–31.23]. More than half (56%, 208/374) had attained post-primary education (Table 1). The households comprised an average of 4 people. The majority of the cooking, 90% (337/374), was done by the spouse of the household head.

**Table 1:** Socio-demographic characteristics of respondents of Kisneyi-Mugunu slum, Fort Portal City, Uganda

Variable	Category	Frequency *N* = 374 (%)
**Age category**	Below 20	43 (11.5)
	21–30	195 (52.14)
	31–40	98 (26.2)
	41–50	21 (5.61)
	Above 50	17 (4.55)
**Level of education**	No formal education	27 (7.22)
	Primary	139 (37.17)
	Secondary	181 (48.40)
	Tertiary	27 (7.22)
**Religion**	Anglican	100 (26.74)
	Catholic	157 (41.98)
	Muslim	73 (19.52)
	Pentecostal	31 (8.29)
	Seventh Day Adventist	13 (3.48)
**Marital status**	Living with partner	168 (44.92)
	Single	134 (35.83)
	Married	60 (16.04)
	Widowed	7 (1.87)
	Divorced	5 (1.34)
**Person who usually does the cooking**	Spouse of the household head	337 (90.11)
	Another relative	21 (5.61)
	Maid/ House helper	15 (4.01)
	Do not cook at all	1 (0.27)

### Cooking characteristics among households

More than three quarters (89%, 332/374) of the respondents used charcoal as the main type of fuel, while less than 1% used LPG or electricity (Table 2). Respondents, on average, spent USD$0.6 (SD ± 0.02) on fuel daily. Above half, 52% (194/374) found the daily cost of the fuel acceptable, while 29% (107/374) reported the daily price as not affordable. The households cooked an average of 2 meals a day, and about 4.5 h (SD ± 1.63), 95%CI [4.07 – 4.40), range [0 – 13] were spent cooking per day. More than half (63%, 237/374) did not have a separate room used as a kitchen (Table 2). Half 52% (194/374) did their cooking outdoors, while 16% (60/374) usually cooked indoors. Cooking in a separate building outside the house was reported by 32% (120/374) of the households. Over half, 57% (190/332) that used charcoal as the main type of fuel reported cooking outdoors, while 94% (31/33) of those that used wood reported cooking from a separate building outside the house.

**Table 2:** Cooking characteristics among households in Mugunu slum, Fort Portal City, Uganda

Variable	Category	Frequency *N* = 374 (%)
**The main type of cooking fuel**	Charcoal	332 (88.77)
	Electricity	1 (0.27)
	Kerosene	3 (0.80)
	LPG/cylinder gas	2 (0.53)
	Straw/shrubs/grass	3 (0.80)
	Wood	33 (8.82)
**Affordability of fuel**	Affordable	194 (51.87)
	Not affordable	107 (28.61)
	Very Affordable	73 (19.52)
**Separate room as a kitchen**	Yes	137 (36.63)
	No	237 (63.37)
**Location of cooking area**	Inside the house	60 (16.04)
	Outdoors	194 (51.87)
	Separate building	120 (32.09)
**Location of kitchen windows**	Not close to the main entrance door	278 (74.33)
	Close to the main entrance door	96 (25.67)
**Fuel storage area**	Inside the house	164 (43.85)
	Outdoors	73 (19.52)
	Separate building	137 (36.63)
**Adequacy of cooking area ventilation**	Not adequate	149 (39.84)
	Adequate	225 (60.16)
**Traditional cook stove**	Yes	311 (83.16)
	No	63 (16.84)
**State of repair of the traditional stove (*n* = 311)**	Good	260 (83.60)
	Not good	51 (16.40)
**Improved cook stove**	Yes	92 (24.60)
	No	282 (75.40)
**State of repair of Improved cook stove (*n* = 92)**	Good	83 (90.22)
	Not good	9 (9.78)
**Fuel biomass storage area protected from water ((*n* = 368)**	Yes	236 (64.13)
	No	132 (35.87)
**Fuel biomass damp (*n* = 368)**	No	211 (57.34)
	Yes	157 (42.66)

**Fig. 2:**
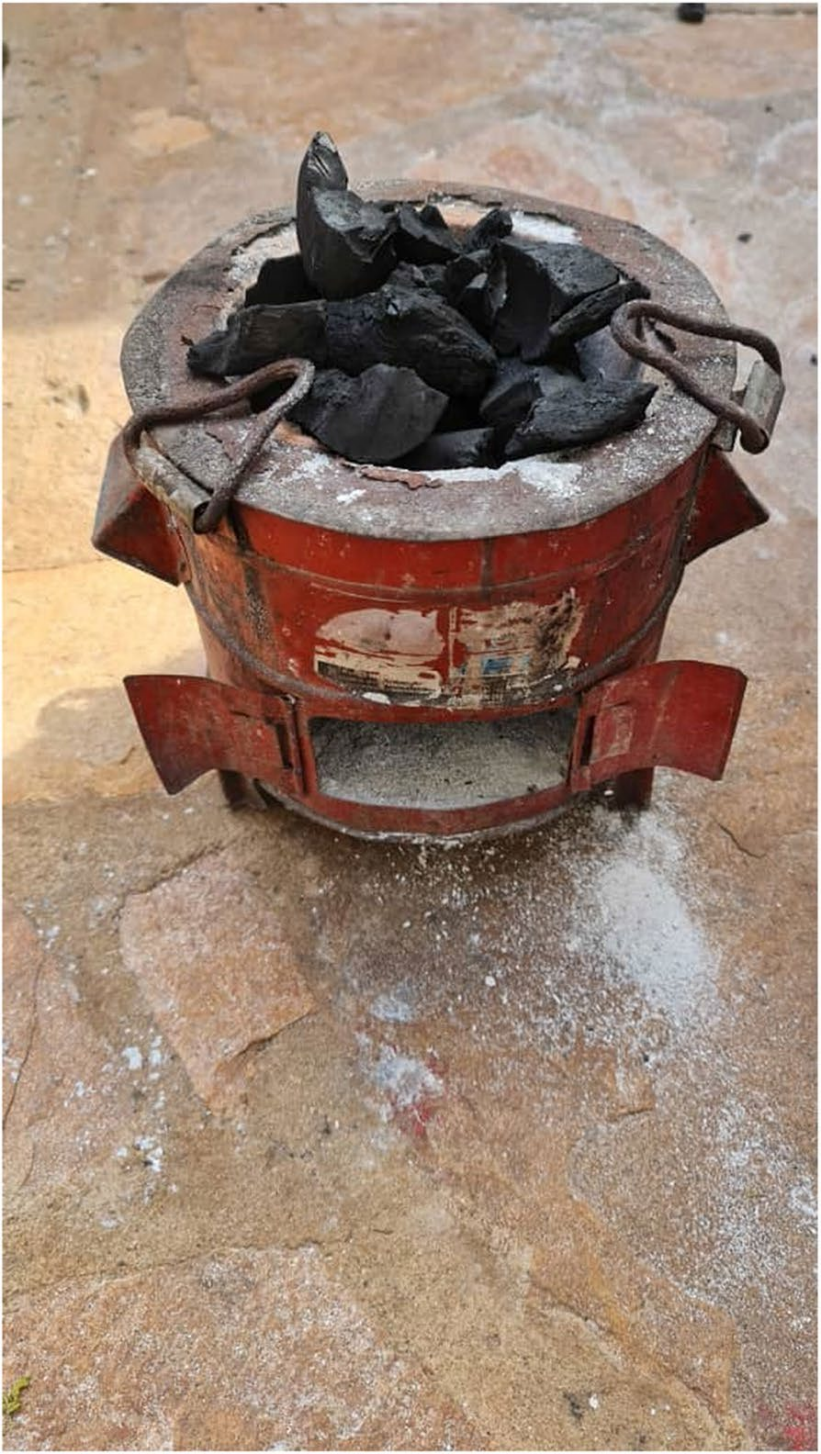
Traditional cook stove

The majority (74%, 278/374) of the households did not have a window close to the main entrance door. However, adequate cooking area ventilation was observed for 60% (225/374) of the households (Table 2). For those who cooked outdoors, the average cooking distance from the house’s main entrance was 3.14 ± 0.17 0 m. The cooking time in the study was generally between 8:00 AM and 6:00 PM. Traditional portable and lightweight charcoal cookstove made of metal with a ceramic liner and one fire per pot batch-fed were used by most households, 83% (311/374) and of these, 84% (265/311) were in a good work condition (\* MERGEFORMAT Fig. 2).

Almost half (44%, 164/374) of the respondents stored fuel indoors, and 37% (137/374) stored the fuel in a separate building outside the house. However, only 64% (236/368) reported that the biomass fuel was protected from rainwater, while 157/368 (43%) were found using damp biomass fuel (Table 2).

The average temperature was 30 °C, while the average humidity was 54% for the cooking area. For the living area, the average temperature was 31 °C, and the average humidity was 52%. During the week of this study, a 24 h average PM_2.5_ of 69.62 µg/m^3^ was reported 382 m away in the neighbouring Rwengoma village [23]. The average ambient temperature range was 16–24.8 °C; average precipitation was 5 mm/d; average humidity was 80.5%; and average wind speed range was 0.2–2.2 m/s [40].

### Predictors of PM_2.5_ and CO concentration

During the cooking time, the mean PM_2.5_ concentrations for the cooking and living area were 175.93 ± 12.49 µg/m^3^ and 124.29 ± 7.95 µg/m^3^, respectively (Table 3). The mean CO concentration during the cooking time was 41.22 ppm and 15.23 ppm for the cooking and living areas, respectively. From an unpaired t-test, it was found that there was a statistically significant difference between PM_2.5_ and CO concentration in the cooking and living area (t = 18.14, *p* ≤ 0.05 and t = 5.77 *p* ≤ 0.05).

**Table 3:** Mean concentration of PM_2.5_ and CO for the cooking and living area in households in Mugunu slum, Fort Portal City, Uganda

	**Cooking area PM**_**2.5**_	**Living area PM**_**2.5**_	**Cooking area CO**	**Living area CO**
Mean	175.93	124.29	41.22	15.53
Std. Err	12.49	7.95	3.31	1.70
SD	241.51	153.76	64.07	32.92
[95% CI]	151.37—200.49	108.66—139.92	34.71—47.74	12.18—18.87
Range	0—1146	0—999.9	0—456	0—267
99% IQ	999.9	873.6	334	213

Cooking in a separate building outside the house resulted in the highest pollution with PM_2.5_ and CO of 176.13 µg/m^3^ and 46.86 ppm, respectively (Table 4). Cooking outdoors also presented high levels of PM_2.5_ (162.58 µg/m^3^) and CO (44.37 ppm). Cooking with all fuel types showed high concentrations of PM_2.5_ and CO in this informal settlement’s cooking and living areas (Table 4).

**Table 4:** Distribution of PM_2.5_ and CO concentration by cooking area location in households in Mugunu slum, Fort Portal City, Uganda

	**PM **_**2.5**_** (µg/m**^**3**^**)**	**CO (ppm)**
**Location of cooking area**	Mean (SD)	Mean (SD)
Indoors (*n* = 60)	162.58 ± 11.79	44.37 ± 4.57
Outdoors (*n* = 194)	166.66 ± 6.72	39.27 ± 2.49
Separate building outside the house (*n* = 120)	176.13 ± 9.45	46.86 ± 3.12

There was a difference in living area concentration of PM_2.5_ and CO by location of cooking area (Table 3). Outdoor cooking resulted in higher PM_2.5_ concentrations than indoor cooking, however, it showed lower CO concentration. Cooking in a separate building outside the house showed lower PM_2.5_ and CO concentration than indoor and outdoor cooking.

At multivariate analysis cooking outdoors was associated with a 0.112 increment in PM_2.5_ concentrations in the cooking area (β_cooking outdoors_ = 0.112 [95% CI: -0.069, 1.614; *p* = 0.033]) (Table 5). Considering the majority of households cooked outdoors in this study, further analysis on cooking outdoors revealed that cooking with less polluting and moderately polluting fuel was associated with a 1.77 (β^2^_cooking outside* less polluting_ = -1.77(-3.355, -0.186) and 0.934 (β^2^_cooking outside* moderately polluting_ = -0.934 (-1.736, -0.133) decrement in PM_2.5_ respectively (Table 5). However, cooking with moderately polluting fuel was associated with a 0.719 increment in CO concentrations (β_moderately polluting_ = 0.718 [95% CI: 0.084, 1.352; *p* = 0.027]) in the living room (Table 6).

**Table 5:** Adjusted regression coefficient for predictors of PM_2.5_ concentrations in the cooking and living area in households in Mugunu slum, Fort Portal City, Uganda

Variable	Cooking area		Living area	
	**Coefficient (95% CI)**	***P*-value**	**Coefficient (95% CI)**	***P*-value**
Adequate ventilation				
No	ref		ref	
Yes	-0.027(-0.213, 0.159)	0.78	-0.062(-0.224, 0.099)	0.47
Window close to the door				
No	ref		ref	
Yes	0.176 (-0.022, 0.374)	0.08	0.043 (-0.131, 0.215)	0.63
Damp fuel				
No	ref		ref	
Yes	0.112(-0.064, 0.289)	0.21	0.048 (-0.105, 0.202)	0.53
**Cooking outside**				
No	ref		ref	
Yes	0.112(0.069, 1.614)	^*^ 0.03	-0.024 (-0.201, 0.152)	0.76
Type of fuel category				
Less polluting	0.272(-0.800, 1.345)	0.62	0.022( -0.600, 0.645)	0.95
Moderately polluting	-0.377(-1.115, 0.362)	0.32	0.031( -0.217, 0.27	0.81
Highly polluting	ref		ref	
Cooking outside ^*^ Type of fuel category				
Less polluting	-1.770(-3.355, -0.186)	^*^ 0.03	-	-
Moderately polluting	-0.934 (-1.736, -0.133)	^*^ 0.02	-	-
Highly polluting	ref		-	-

^*^ *p*-value less than 0.05

Considering a 95% CI, a *p*-value ≤ 0.05 was considered to be statistically significant in this study

**Table 6:** Adjusted regression coefficient for variables associated with the concentration of CO in the cooking and living area in households in Mugunu slum, Fort Portal City, Uganda

Variable	Cooking area		Living area	
	**Coefficient (95% CI)**	***P*-value**	**Coefficient (95% CI)**	***P*-value**
Adequate ventilation				
No	ref		ref	
Yes	-0.124( -0.479,0 .231)	0.49	-0.258(-0.619, 0.102)	0.16
Window close to the door				
No	ref		ref	
Yes	0.283( -0.086, 0.652)	0.13	-.019( -0.401, 0.363)	0.92
Damp fuel				
No	ref		ref	
Yes	-0.086( -0.421, 0.249)	0.61	-0.0669(-0.402, 0.269)	0.69
Cooking outside				
No	ref		ref	
Yes	0.493( -1.454, 2.441)	0.62	-0.207(-0.596, 0.182)	0.30
Type of fuel category				
Less polluting	1.697( -0.589, 3.982)	0.15	1.314(-0.034, 2.663)	0.06
Moderately polluting	0.427( -1.474, 2.328)	0.66	0.718( 0.084, 1.352)	^*^ 0.03
Highly polluting	ref		ref	
Cooking outside ^*^ Type of fuel category				
Less polluting	-1.608( -5.127, 1.912)	0.37	-	-
Moderately polluting	-0.129( -2.116, 1.858)	0.90	-	-
Highly polluting	ref		-	-

*p*-value less than 0.05

Considering a 95% CI, a *p*-value ≤ 0.05 was considered to be statistically significant in this study

## Discussion

This study aimed to assess household characteristics that predict the concentrations of PM_2.5_ and CO within households in an informal urban settlement in the newly created city of Fort Portal city, Uganda. The main type of cooking fuel used by the households was charcoal. The average ambient PM_2.5_ concentration of the neighbouring village was above the WHO Air Quality. Cooking outdoors  was associated with higher PM_2.5_ concentrations in the cooking area. However, cooking outdoors using LPG and charcoal showed a reduction of PM_2.5_ concentrations compared to highly polluting fuels of wood, straw/ shrubs, and grass. Cooking with charcoal was associated with higher CO concentration in the living space.

In this study, charcoal was the main type of fuel used for cooking. Charcoal is a readily available and accessible fuel as this district is surrounded by forests that serve as a wood source for charcoal burning. Similar studies conducted in informal settings have found a smaller proportion of households using charcoal or wood at the household level [41–43]. However, this study’s findings are comparable to results from a nearby city of Mbarara, Uganda, and Avenor in Accra, Ghana, where charcoal was reported to be the most commonly used cooking fuel [27, 44]. The burning of charcoal biomass has environmental and health effects. The demand for charcoal encourages deforestation that destroys habitats of vital ecosystems leading to a reduction in ecosystem services, including tourism and climate change. Combustion of charcoal releases particulate matter and volatile organic compounds, including PM_2.5_ and CO, in the cooking and living area, as was observed in this study.

The ambient PM_2.5_ in the neighbouring Rwengoma village was higher than the WHO Air Quality limits of 15 μg/m^3 ^[45] and 46.944 µg/m3 reported during informal wet season coastal settlements of Lagos, South-western Nigeria [46]. The higher concentration of ambient PM_2.5_ could be due to fuel filling stations, vehicular traffic emissions, auto mechanic garages [22, 47–49], open burning of solid waste, high-density built environment and anthropogenic activities [50], and the predominant use of solid biomass fuels. High ambient air quality is associated with low IAQ [49] primarily for solid fuel-burning communities. In addition, ambient air pollutants contribute to IAP by infiltrating pollutants and dispersal through open windows and doors [49].

This study observed that only 1% of the households used LPG and electricity. Although all households were electrified, the inhibitive recurrent domestic consumer cost of electricity at USD$0.21 per 1 kWh [51] and the high cost of USD$14 for a 6 kg LPG cylinder [52] may have limited the usage of electricity and LPG, respectively for cooking in this community. However, the households that used less polluting fuels of electricity and LPG also had high mean PM_2.5_ concentrations indoors. This study finding indicates the possible infiltration of ambient PM_2.5_ and CO from neighbouring pollutant sources, including proximal households that use solid biomass for cooking [53]. This study observed that the usage of LPG and electricity by 1% of households did not reduce PM_2.5._ However, the finding may imply that the switch to less polluting fuels has to happen for a significant proportion of the neighbourhood for the protective effect of cleaner energy against PM_2.5_ and CO to be realised.

Using moderately polluting fuel (charcoal) was associated with a higher CO concentration in the living area. Incomplete combustion of charcoal in the traditional cookstoves may have resulted from the accumulation of CO, especially in the poorly ventilated living spaces. Other studies have shown an association between charcoal combustion and increased indoor CO [54, 55]. Exposure to indoor CO can result in the accumulation of toxic concentrations with mild and short-term exposure resulting in nausea, headaches, dizziness, impaired psychomotor function, loss of balance, fatigue and respiratory symptoms [8, 56, 57], while long-term exposure to CO could result into loss of consciousness and death [54].

Cooking outdoors was associated with a 0.112 unit increase in PM_2.5_. Most households cooked outside to avoid getting smoke inside their houses as they lacked space designated for cooking in their single or double-roomed structures. Cooking outdoors results in the dispersal of airborne particles away from the cooking area by ambient air currents. However, ambient PM_2.5_ particles that were observed may also have contributed to the PM_2.5_ in the outdoor cooking areas. Cooking location is an identified factor that influences the average concentrations of smoke in the cooking areas [58, 59]. For this study, the positive benefits of cooking outdoors may have been negated by the high ambient air PM_2.5_.

Although cooking outdoors in this informal urban settlement was associated with higher PM_2.5_, using less or moderately polluting cooking fuel resulted in lower PM_2.5_ concentrations (less polluting cooking fuel ˂ moderately polluting cooking fuel). When using traditional cookstoves, incomplete combustion of solid biomass fuels occurs due to difficulty in mixing of the solid fuel and air during burning, unlike for LPG (gas) and kerosene (liquid), leading to the release of a significant proportion of products of incomplete combustion PM_2.5_ [10, 60]. The lower concentration of PM_2.5_ concentration with the type of cooking fuel is similar to a study conducted in rural Malawi that assessed the effect of cooking location and type of cooking fuel on the level of PM_2.5_ [61]. Using less polluting fuels during outdoor cooking in informal settings may contribute to a lower concentration of PM_2.5_ in the cooking area, emphasising the need to promote cleaner cooking energy.

This study determined the cooking time PM_2.5_ and CO concentrations of the cooking and living areas for most households in this informal urban settlement. The cooking period presented the most eminent danger as fuel sources are actively burning with the highest expected concentration of incomplete combustion products of PM_2.5_ and CO.

## Conclusions

The cooking and the living areas had high concentrations of PM_2.5_ and CO during the cooking time. Cooking with charcoal resulted in higher CO in the living area. Furthermore, cooking outdoors did not have a protective effect against PM_2.5,_ and ambient PM_2.5_ exceeded the WHO Air quality limits. This was due to the predominant usage of charcoal for cooking fuel coupled with the congestion of households, abundant fuel filling stations and poor ambient air quality, which impact the indoor air quality in this community. Interventions to improve the indoor air quality in informal settlements need to promote a switch to cleaner cooking energy for all households in the neighbourhood for the benefit of reduction in indoor PM_2.5_ and CO concentrations to be realised.

## Supplementary Information

**Additional file 1.** 

## Data Availability

The datasets used and analysed during the current study are available from the corresponding author on reasonable request.

## Abbreviations

ALRI: Acute lower respiratory infections
CO: Carbon monoxide
DALYs: Disability-adjusted life years
HAP: Household air pollution
IAP: Indoor air pollution
LMICs: Low- and middle-income countries
LPG: Liquefied petroleum gas
NCDs: Non-communicable diseases
NO2: Nitrogen dioxide
PM: Particulate matter
VAT: Value added tax
WHO: World Health Organization
