# Supplementary material for: Use of biomass fuels predicts indoor particulate matter and carbon monoxide concentrations; evidence from an informal urban settlement in Fort Portal city, Uganda
Source: BMC Public Health. 2022 Sep 12;22:1723. doi: 10.1186/s12889-022-14015-w (PMC9464485; doi:10.1186/s12889-022-14015-w)
Supplement: Supplementary file 1 — Additional file 1. [file 12889_2022_14015_MOESM1_ESM.docx]

### **Appendix 3: OBSERVATON QUESTIONNAIRE**

**STUDY TITLE: Use of biomass fuels predicts indoor particulate matter and carbon monoxide concentrations; evidence from an informal urban settlement in Fort Portal city, Uganda**

| **Structure& household number:** |  | **Date:** |  |
| --- | --- | --- | --- |
| **Interviewers Name** |  | | |
| **Please Read Consent form to the participant and put your initials** | | I have read the consent form to the participant | |

**SECTION A:** PREDICTORS OF INDOOR AIR QUALITY

|  |  |  |  | |
| --- | --- | --- | --- | --- |
| 1a) | Is the cooking done inside the house? | Yes | No | |
| 1b) | Is the cooking done outside the house? | Yes | No | |
| 1c) | Is the cooking done in a separate building outside the house? | Yes | No | |
| 1d) | If cooking is done outside the house, how far from the main entrance? (in metres) |  |  | |
| 2a) | Does the living space have through ventilation? | Yes | No | |
| 2b) | Does the living space have cross ventilation? | Yes | No | |
| 2c) | Does the cooking area have adequate ventilation? | Yes | No | |
| 3 | Are the kitchen windows located close to the door? | Yes | No | |
| 4a) | Is a tradition cook stove used or cooking? | Yes | No | |
|  | Is it in good state of repair? | Yes | No | |
| 4b) | Is an improved cook stove for cooking? | Yes | No | |
|  | Is it in good state of repair? | Yes | No | |
| 4c) | If none of the above is used, what type of cook stove is used in the household? | Yes | No | |
| 4d) | Is it in good state of repair? | Yes | No | |
| 5a) | Is the fuel source stored inside the house | Yes | No | |
| 5b) | Is the fuel source stored outside the house | Yes | No | |
| 5c) | Is the fuel source stored in separate building outside the house | | Yes | No |
| 6a) | Is the storage area well protected from water running into the fuel source?  (look for water around the fuel source or possible flow in of water) | | Yes | No |
| 6b) | Is the fuel source damp? | | Yes | No |
| 6c) | Did it rain in the last one week in this village before today’s visit?  (you can ascertain from the respondent) | | Yes | No |
| 7a) | Are the children with the mother/caretaker as she does the cooking? | | Yes | No |
| 7b) | How far from the cooking are the children?  ( record in metres) | | ……….metres | |

**THANK YOU SO MUCH FOR YOUR TIME AND THE VALUABLE INFORMATION**
